# Preservation of the Native Esophagus in Long-Gap Esophageal Atresia: Case Presentation

**DOI:** 10.7759/cureus.106915

**Published:** 2026-04-12

**Authors:** Silvia N Suarez Mantilla, Enna Melissa Rodriguez Cardenas, Angie Daniela Lizarazo Castellanos, Sergio Zavaleta Hernandez, Martha Lucia Africano Leon

**Affiliations:** 1 Pediatrics, Industrial University of Santander, Bucaramanga, COL; 2 Pediatrics, University of Santander, Bucaramanga, COL; 3 Paediatrics, University of Santander, Bucaramanga, COL; 4 Paediatrics and Child Health, San Luis Maternal and Child Clinic, Bucaramanga, COL; 5 Paediatrics, Industrial University of Santander, Bucaramanga, COL; 6 Paediatrics, San Luis Maternal and Child Clinic, Bucaramanga, COL

**Keywords:** colombia, gastro esophageal disease, how to manage tracheoesophageal fistula, long-gap esophageal atresia, surgical anastomosis, tracheoesophageal fistula, tracheoesophageal fistula repair

## Abstract

Esophageal atresia type 1 is a rare birth defect that involves the loss of esophageal continuity without the presence of a tracheoesophageal fistula. In prenatal testing, polyhydramnios and the absence of a gastric bubble were observed. At birth, sialorrhea, cyanosis, and difficulty progressing the orogastric tube were noted. Although minimally invasive surgery with preservation of the native esophagus represents an innovative strategy to reduce morbidity and improve quality of life, it hasn't yet become the standard management due to its technical requirements and low volume of global cases. Three cases of patients with type I esophageal atresia with a long esophageal gap are presented, where the native esophagus was preserved after serial measurements of the esophageal ends with gastrostomy and thoracoscopic surgery or delayed open reconstruction without an esophagostomy in the first two months of life. Preserving the native esophagus in the correction of esophageal atresia is a viable alternative that neonatal referral centers can consider and replicate.

## Introduction

Esophageal atresia (EA) is a congenital malformation characterized by a discontinuous esophageal lumen, with or without tracheoesophageal fistula. Type A (also called Type 1) is rare, occurring in one in 3,500-4,500 newborns; it features two blind-ending esophageal pouches without a fistula [1,2]. It often involves a long gap that is commonly defined as an EA with no air in the abdomen and/or a gap ≥3 vertebral bodies or ≥2-3 cm between esophageal ends, making repair more challenging than the common Type C [1,3-5]. Prenatal diagnosis may suggest polyhydramnios with an absent gastric bubble. Postnatally, signs include sialorrhea, respiratory distress, cyanosis, and failure to pass an orogastric tube. Radiographs show no distal intestinal gas and a coiled tube in the proximal pouch. Historically, wide gaps prompted neonatal cervical esophagostomy and gastrostomy for feeding and weight gain, delaying reconstruction (e.g., with stomach, colon, or jejunum) until aged one-year or later. Despite advances in neonatal care and early surgery improving survival, long-term complications after reconstruction include dysphagia, Barrett's esophagus, chronic chest pain, and nutritional deficits [2-4]. Recent progress in neonatal surgery, parenteral nutrition, and intensive care has enabled native esophagus preservation via staged procedures, even in Type A cases, with lifelong follow-up [2-4].

## Case presentation

Case one

**Figure 1:**
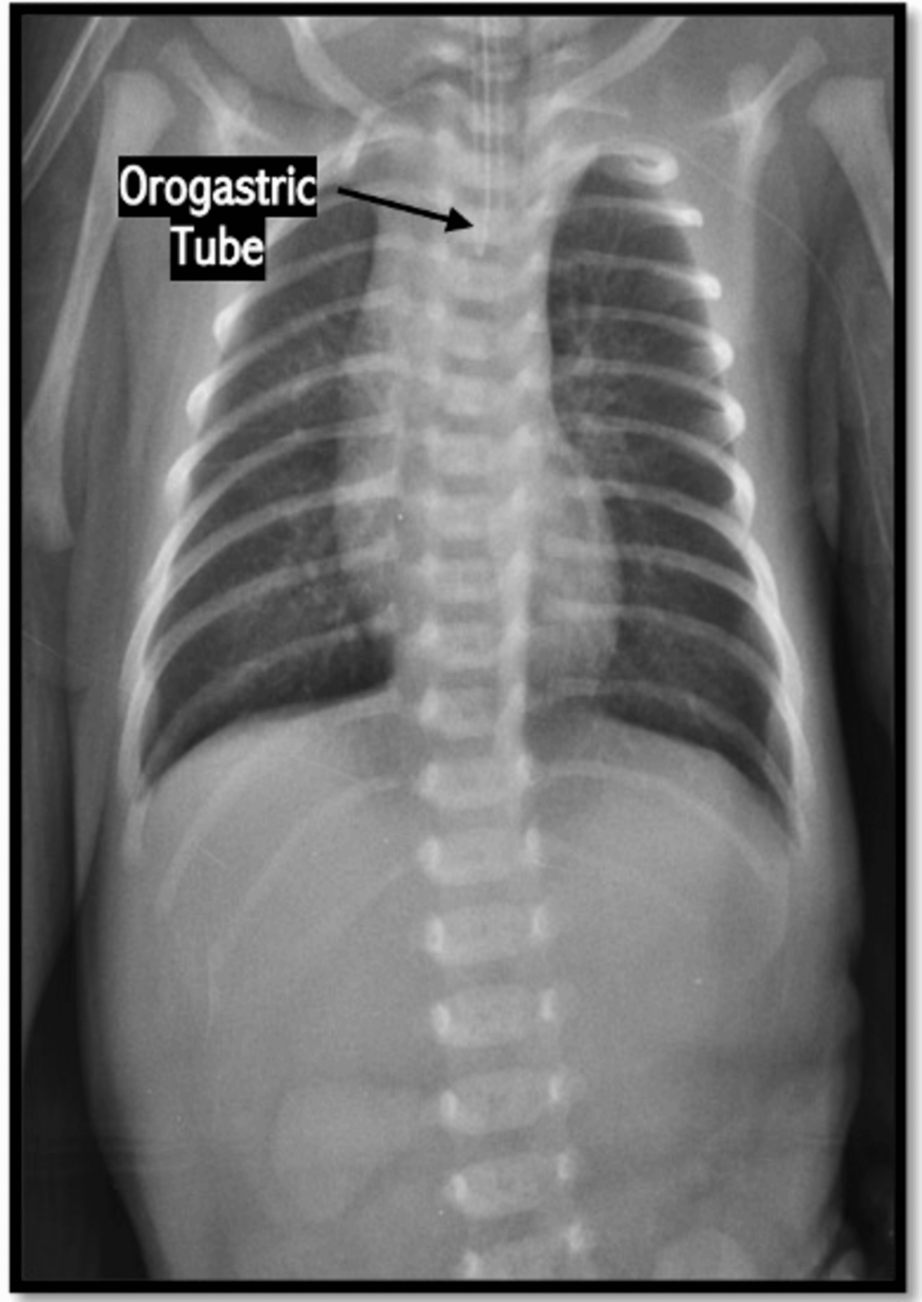
Absence of gastric bubble Thoracoabdominal X-ray showing an orogastric tube coiled at the cervical esophageal level with absence of a gastric bubble. The study was performed in a newborn presenting with sialorrhea and respiratory distress requiring mechanical ventilation after unsuccessful advancement of the orogastric tube. Findings are suggestive of esophageal atresia.

A term male newborn (37.3 weeks of gestation) with a birth weight of 2,585 g was delivered by cesarean section due to severe polyhydramnios. After birth, the patient presented with sialorrhea and respiratory distress requiring mechanical ventilation. Chest radiography demonstrated an orogastric tube coiled at the cervical level and the absence of a gastric bubble (Figure 1), findings suggestive of pure esophageal atresia.

On the third day of life, a laparoscopic gastrostomy was performed. During the procedure, fluoroscopy was used to measure the distance between the esophageal ends in real time by introducing bile duct dilators through the oral cavity and the gastrostomy site. The gap measured approximately seven vertebral bodies without tension and three vertebral bodies under maximal tension.

The patient was subsequently managed with enteral feeding through the gastrostomy tube and continuous aspiration of the proximal esophageal pouch using a double-lumen tube for secretion control and irrigation. A second measurement performed on day 14 of life demonstrated a reduction of the gap to approximately 1.5 vertebral bodies under tension.

On day 34 of life, with a weight of 3,350 g, thoracoscopic primary esophageal anastomosis under tension was performed, preserving the native esophagus. On postoperative day five, the patient developed an anastomotic leak with mediastinitis, requiring surgical reintervention for revision of the anastomosis. The anastomosis was preserved, and broad-spectrum antibiotic therapy was administered for seven days.

A postoperative esophagram confirmed restoration of esophageal continuity, allowing initiation of enteral feeding. However, the clinical course was later complicated by nosocomial sepsis, leading to death at three months of age.

Case two

A term female newborn (38 weeks of gestation) with a birth weight of 3,135 g was delivered by cesarean section due to severe polyhydramnios. At birth, the patient presented with sialorrhea and respiratory distress requiring mechanical ventilation. Failure to advance the orogastric tube prompted chest radiography, which demonstrated the tube coiled at the cervical level with absence of a gastric bubble, findings suggestive of pure esophageal atresia.

**Figure 2:**
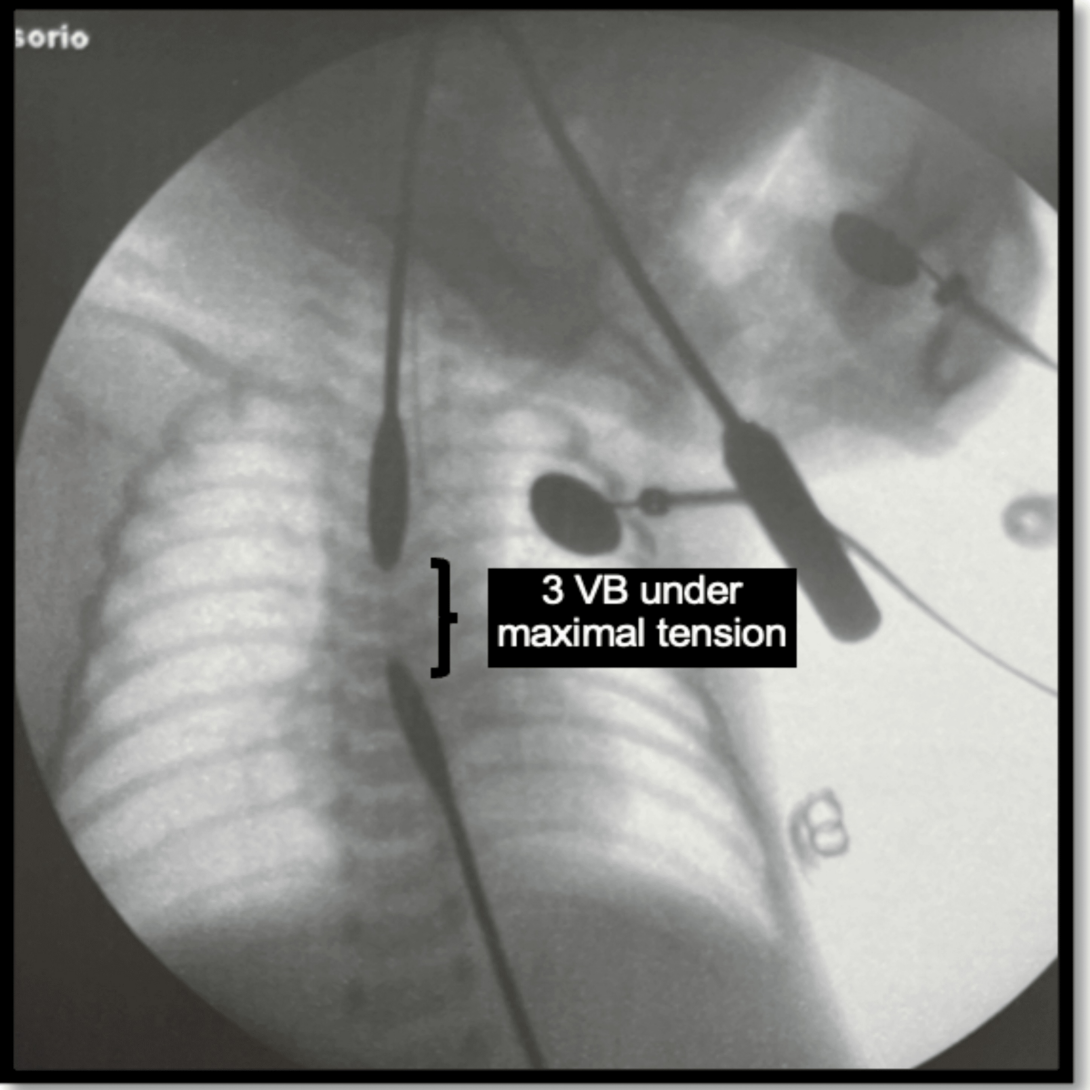
First fluoroscopic measurement of the esophageal gap Fluoroscopic assessment performed on day 13 of life in Patient two demonstrated an esophageal gap measuring approximately three vertebral body lengths under maximal traction. VB: vertebral bodies.

A laparoscopic gastrostomy was initially planned; however, the procedure was postponed due to early neonatal sepsis. On day 13 of life, fluoroscopic measurement of the esophageal gap was performed, demonstrating a distance of five vertebral bodies without tension and three vertebral bodies under maximal traction (Figure 2). A gastrostomy was subsequently created, and continuous aspiration of the proximal esophageal pouch was maintained using a double-lumen tube.

On day 39 of life, with a body weight of 3,200 g, right thoracotomy with primary esophageal anastomosis under tension was performed, preserving the native esophagus. Postoperative esophagram demonstrated satisfactory esophageal continuity without evidence of leakage, allowing initiation of enteral feeding.

At the six-month outpatient follow-up, the patient was tolerating full oral feeding with adequate weight gain and no longer required gastrostomy support.

Case three

**Figure 3:**
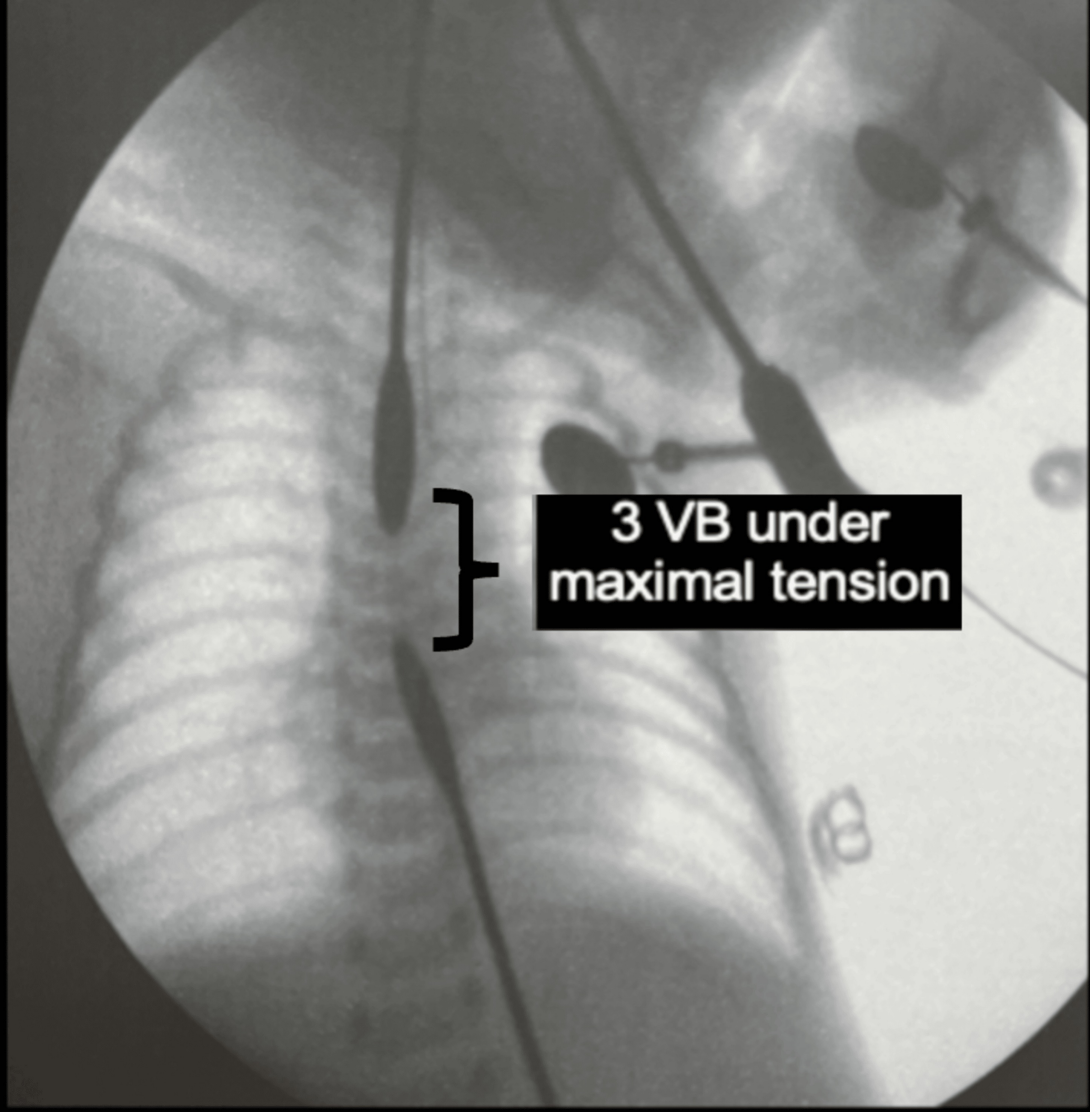
Fluoroscopic measurement of the esophageal gap in case three Fluoroscopy demonstrating an esophageal gap of approximately three vertebral body lengths under maximal traction. VB: vertebral bodies.

A preterm male newborn (34 weeks of gestation) with a birth weight of 2,284 g was delivered by cesarean section due to severe polyhydramnios, with prenatal suspicion of esophageal atresia and trisomy 21. At birth, the patient developed sialorrhea and respiratory distress requiring non-invasive mechanical ventilation for two days. Failure to advance the orogastric tube prompted chest radiography, which demonstrated the tube coiled at the cervical level with the absence of a gastric bubble. A gastrostomy was performed at the birth hospital on day three of life, and the patient was referred to our institution at 13 days of age. The first fluoroscopic measurement of the esophageal gap was obtained on day 18 of life, demonstrating a distance of seven vertebral bodies without tension and four vertebral bodies under maximal traction. The patient was managed with enteral feeding through the gastrostomy and continuous aspiration of the proximal esophageal pouch using a double-lumen tube. A second measurement performed at 38 days of life demonstrated a reduction of the gap to approximately 2.5 vertebral bodies under tension (Figure 3). Definitive repair was initially deferred due to suspected late neonatal sepsis and inadequate weight gain.

On day 70 of life, right thoracotomy with primary esophageal anastomosis under tension was performed, preserving the native esophagus. Approximately two weeks postoperatively, the patient developed an anastomotic stricture requiring initiation of an esophageal dilation program. However, follow-up was interrupted, and the patient was readmitted three months later with critical esophageal stenosis requiring surgical reintervention to restore luminal patency.

At present, follow-up, the patient tolerates oral feeding, with decreasing dependence on gastrostomy for nutritional support (Table 1).

**Table 1:** Characterization of cases of native esophageal preservation in wide-gap esophageal atresia without fistula.

	Clinical Case One	Clinical Case Two	Clinical Case Three
Sex	Male	Female	Male
Birth weight (grams)	2.585g	2.220g	2.284g
Weight on the day of surgery (grams)	3.350g	3.200g	3.340g
weight at surgery (grams)	37.3 weeks	38 weeks	34 weeks
Age at surgery	34 days of life	39 days of life	70 days of life (Deferred due to sepsis)
First measurement	On day 3 of life: With tension of the 3 vertebral bodies. Without tension, 7 vertebral bodies.	On day 13 of life: With tension of 3 vertebral bodies. Without tension, 5 vertebral bodies.	On day 18 of life: With tension of 4 vertebral bodies. Without tension, 7 vertebral bodies.
Second measurement	-	-	At 38 days of life: With tension of 2.5 vertebral bodies.
Associated anomalies	Bilateral cryptorchidism, Ductus Arteriosus, Single umbilical artery	Interatrial Communication	Trisomy 21, Hypothyroidism
Immediate post-surgical complications	Leak mediastinitis resolved.	S. epidermidis bacteremia. Peristomal cellulitis.	Multilobar pneumonia and septic shock.
late complications	Septic shock	-	Stenosis of the distal ⅓ of the correction.
Outcomes	Death due to septic shock.	At 3 months post-surgery, in ambulatory follow-up, fed orally, good weight gain, in gastrostomy withdrawal plan.	At 6 months post-surgery ambulatory follow-up, fed orally, good weight gain, in gastrostomy withdrawal plan.

## Discussion

In Colombia, this is the first case report of esophageal atresia type I with preservation of the native esophagus. As a result of the distance between both esophageal ends, surgery cannot be performed in the first days of life. These three cases illustrate the clinical management and outcomes of Type I EA with long-gap using a strategy of delayed primary anastomosis with preservation of the native esophagus.

The cases demonstrate the feasibility of applying internationally accepted surgical strategies in a local setting. Delayed primary anastomosis, performed after a period of spontaneous growth of the esophageal ends, allowed restoration of esophageal continuity in all three patients. Serial measurement of the esophageal gap using fluoroscopy and vertebral body references enabled objective monitoring of esophageal growth and guided the timing of definitive repair [3-11]. This experience suggests that complex neonatal surgical approaches commonly reported in high-resource centers can also be successfully implemented in specialized centers in Colombia.

Several surgical strategies have been described for long-gap esophageal atresia, each with specific advantages and limitations. These include delayed primary anastomosis, traction techniques (such as the Foker technique or internal traction), and esophageal replacement using gastric or colonic conduits [8-12].

More recently, international collaborative groups such as the International Network of Esophageal Atresia (INoEA), European Reference Network for Rare Inherited and Congenital (digestive and gastrointestinal) Anomalies (ERNICA), and American Pediatric Surgical Association (APSA), as well as large clinical series, have recommended preservation of the native esophagus through delayed primary anastomosis or traction-based “growth” techniques as the first-line approach when feasible [4-12]. Minimally invasive surgery offers advantages, including improved visualization of the thoracic cavity and more precise manipulation of mediastinal and esophageal structures; however, it requires a significant learning curve and specialized technical expertise from the surgical team [13-16].

Despite similar surgical strategies, the cases highlight variability in clinical outcomes. Two patients achieved preservation of the native esophagus and eventual oral feeding, although complications such as anastomotic leak and postoperative stenosis required dilation or reoperation. One patient died due to nosocomial sepsis, suggesting that mortality in these patients may be more closely related to postoperative complications and hospital-acquired infections than to the technical success of the surgical procedure itself [9-10]. These findings reflect challenges frequently reported in neonatal intensive care units in middle- and low-income countries.

These cases also underscore the importance of multidisciplinary and longitudinal care. Given the low incidence of this condition, large case volumes using this strategy remain limited, making it difficult to draw definitive conclusions. Nevertheless, preservation of the native esophagus appears to be a viable and reproducible alternative that neonatal referral centers may consider adopting.

Successful management required coordinated care involving neonatology, pediatric surgery, gastroenterology, radiology, nutritional support, and genetic evaluation, as esophageal atresia is frequently associated with other congenital anomalies that should be systematically investigated, including those described in the vertebral defects, anal atresia, cardiac defects, tracheoesophageal fistula, renal anomalies, and limb abnormalities (VACTERL) association [17]. In addition, long-term follow-up with surveillance for complications, such as esophageal stenosis and potential need for dilation, is essential [4-12]. The third case illustrates how loss to follow-up may lead to severe complications requiring reintervention, representing an ongoing challenge in healthcare systems where access to specialized care is fragmented.

Finally, these cases highlight systemic barriers that influence outcomes in Colombia. Factors such as the low prevalence of the condition, ongoing surgical training in the management of gastrointestinal malformations (including esophageal atresia), delayed referral from other institutions, and difficulties in maintaining long-term follow-up can significantly affect prognosis. Strengthening prenatal diagnosis, referral networks, infection-control practices in neonatal intensive care units, and structured long-term follow-up programs may improve survival and functional outcomes in children with complex congenital anomalies such as type I esophageal atresia.

## Conclusions

This case series of long-gap Type I esophageal atresia highlights that delayed primary esophageal anastomosis with preservation of the native esophagus remains a relevant management strategy in Colombia. Its success relies on careful patient selection and multidisciplinary optimization, including surgical expertise, perioperative management, infection prevention, and structured long-term follow-up within the healthcare system.
